# Correction: Biathletes present repeating patterns of postural control to maintain their balance while shooting

**DOI:** 10.1371/journal.pone.0316863

**Published:** 2024-12-30

**Authors:** Justyna Michalska, Rafał Zając, Krzysztof Szydło, Dagmara Gerasimuk, Kajetan J. Słomka, Grzegorz Juras

There affiliations for the third and fourth author are incorrect. Krzysztof Szydło is not affiliated with #1 but with #2. Dagmara Gerasimuk is not affiliated with #2 but with #1:

Justyna Michalska^1^, Rafał Zając^1^, Krzysztof Szydło^2^, Dagmara Gerasimuk^1^, Kajetan J. Słomka^1^, Grzegorz Juras^1^

**1** Institute of Sport Sciences, The Jerzy Kukuczka Academy of Physical Education, Katowice, Poland, **2** Silesian University of Technology – Sports Center, Gliwice, Poland
