# Supplementary material for: Biathletes present repeating patterns of postural control to maintain their balance while shooting
Source: PLoS One. 2022 May 3;17(5):e0267105. doi: 10.1371/journal.pone.0267105 (PMC9064080; doi:10.1371/journal.pone.0267105)
Supplement: Attachment — Submitted filename: Response to reviewers.docx [file pone.0267105.s003.docx]

**Editor comment**

**Please review your reference list to ensure that it is complete and correct. If you have cited papers that have been retracted, please include the rationale for doing so in the manuscript text, or remove these references and replace them with relevant current references. Any changes to the reference list should be mentioned in the rebuttal letter that accompanies your revised manuscript. If you need to cite a retracted article, indicate the article's retracted status in the References list and also include a citation and full reference for the retraction notice.**

Answer:

We have carefully checked the list of references. All the articles cited in the current manuscript can be found in the bibliography. We have removed from first version of the manuscript following papers:

•	Soligard T, Schwellnus M, Alonso JM, Bahr R, Clarsen B, Dijkstra HP, et al. How much is too much? (Part 1) International Olympic Committee consensus statement on load in sport and risk of injury. Br J Sports Med. 2016;50(17):1030–41.

•	Donker SF, Roerdink M, Greven AJ, Beek PJ. Regularity of center-of-pressure trajectories depends on the amount of attention invested in postural control. Exp Brain Res. 2007;181(1):1–11.

•	Pincus SM, Gladstone IM, Ehrenkranz RA. A REGULARITY STATISTIC FOR MEDICAL DATA ANALYSIS. J Clin Monit. 1991;7(4):335–45.

•	Richman JS, Moorman JR. Physiological time-series analysis using approximate entropy and sample entropy. AmJ Physiol Hear CircPhysiol. 2000;278(0363-6135 (Print)):H2039–49 (duplicate)

However, to improve our rationale of research, we have added following paper to current manuscript:

•	Rhea CK, Silver TA, Hong SL, Ryu JH, Studenka BE, Hughes CML, et al. Noise and complexity in human postural control: Interpreting the different estimations of entropy. PLoS One. 2011;6(3):1–9.

•	Manor B, Costa MD, Kun H, Newton E, Starobinets O, Hyun GK, et al. Physiological c	omplexity and system adaptability: Evidence from postural control dynamics of older adults. J Appl Physiol. 2010;109(6):1786–91.

•	Delgado-Bonal A, Marshak A. Approximate entropy and sample entropy: A comprehensive tutorial. Vol. 21, Entropy. 2019. p. 1–37

•	Arutyunyan GA, Gurfinkel VS MM. Organization of movements on execution by man f an exact postural task. Biophysics (Oxf) [Internet]. 1969;14(6):1162–7. http://www.sciencedirect.com/science/article/pii/S016073831500044

**Reviewer 1**

We are very grateful for a such an approving review and acceptance for publication. It strongly motivates us to conduct further research.

**Reviewer 2**

**The manuscript is much improved from its initial submission. The introduction is more coherent and on point with the research question. Details in the methods have been clarified/modified to enhance the reader’s understanding of the design. However, the results are still written in an incomplete manner. The discussion is much improved and matches the introduction in a better manner with proper interpretations.**

Answer:

We appreciate this comment. We have provided all necessary changes and corrections to clarify and improve the rationale of our research. We are aware that the previous results section might be misleading and unclear. Therefore, we have revised the entire section, see lines 180-225.

**Reviewer comment:**

**The first sentence (line 182-183) has no dependent variable provided in it or is it in the heading, so it is unclear as to what this finding is relative to. The same pretty much happens in the second paragraph but has SampEn referenced in the middle rather than at the beginning.**

Answer:

Thank you for this comment. We have provided a dependent variable and have changed the “AP” and “ML” headings in “Sample entropy in the anterior-posterior plane” and “Sample entropy in the medio-lateral plane,” respectively.

**Reviewer comment:**

**For the "intragroup comparisons during QS, SP, and AT" section, was the significant finding the main effect of condition or the interaction between condition and group? Some of the results provided here do not make it clear. Some language seems to reference the condition effect while other parts speak about group differences being present. Improvement and clarity needed. Similar issues are present for the ML direction material.**

Answer:

We fully understand the reviewer’s concern. As previously mentioned, we have revised this section, see lines 180-225.

We have also sent our manuscript to Professional English Services. All grammatical corrections have been highlighted in the Revised manuscript with track changes
